# Analysis of genetic diversity and selection characteristics using the whole genome sequencing data of five buffaloes, including Xilin buffalo, in Guangxi, China

**DOI:** 10.3389/fgene.2022.1084824

**Published:** 2023-01-09

**Authors:** Zhefu Chen, Min Zhu, Qiang Wu, Huilin Lu, Chuzhao Lei, Zulfiqar Ahmed, Junli Sun

**Affiliations:** ^1^ Guangxi Key Laboratory of Livestock Genetic Improvement, Animal Husbandry Research Institute of Guangxi Zhuang Autonomous Region, Nanning, China; ^2^ College of Animal Science and Technology, Northwest A&F University, Xianyang, China; ^3^ Faculty of Veterinary and Animal Sciences, University of Poonch Rawalakot, Rawalakot, China

**Keywords:** Xilin buffalo, genomic diversity, population structure, genetic signatures, whole genome sequencing

## Abstract

Buffalo is an economically important livestock that renders useful services to manhood in terms of meat, milk, leather, and draught. The Xilin buffalo is among the native buffalo breeds of China. In the present study, the genetic architecture and selection signature signals of Xilin buffalo have been explored. Correlation analysis of the population structure of Xilin buffalo was conducted by constructing NJ tree, PCA, ADMIXTURE and other methods. A total of twenty-five (*n* = 25) Xilin buffalo whole genome data and data of forty-six (*n* = 46) buffaloes published data were used. The population structure analysis showed that the Xilin buffalo belong to the Middle-Lower Yangtze. The genome diversity of Xilin buffalo was relatively high. The CLR, π ratio, *F*_*ST,*_ and XP-EHH were used to detect the candidate genes characteristics of positive selection in Xilin buffalo. Among the identified genes, most of the enriched signal pathways were related to the nervous system and metabolism. The mainly reported genes were related to the nervous system (*GRM5, GRIK2, GRIA4*), reproductive genes (*CSNK1G2, KCNIP4*), and lactation (*TP63*). The results of this study are of great significance for understanding the molecular basis of phenotypic variation of related traits of Xilin buffalo. We provide a comprehensive overview of sequence variations in Xilin buffalo genomes. Selection signatures were detected in genomic regions that are possibly related to economically important traits in Xilin buffalo and help in future breeding and conservation programs of this important livestock genetic resource.

## 1 Introduction

Domestic buffaloes are predominantly distributed in Asian countries. According to behavior and chromosome karyotype, domestic buffaloes are divided into two types: riverine buffalo (*Bubalus bubalis*, 2 *n* = 50) and swamp buffalo (*Bubalus bubalis* carabanensis, 2n = 48) (Fischer and Ulbrich, 1967). As an important economic livestock species in the world, the important traits e.g., milk production, growth, reproduction, hair color, etc. Have been focused previously as important indicators for selection (Liu et al., 2018). From the year 1999–2019, the number of buffalo increased by about 25.9% which in turn increased milk production by 106% and buffalo beef production by 45% (Di Stasio and Brugiapaglia, 2021). In addition, buffalo is a good drought animal that compensate for about 20%–30% of the agricultural labor force (Michelizzi et al., 2010). Although with the popularization of mechanization, the role of buffalo as a servant has been gradually replaced, it is still the most important source of labor in some remote mountainous areas in southern China. The buffaloes are used for plowing the agricultural land, particularly paddy rice fields. In addition, buffaloes are used in a cart for transporting heavier goods as compared with cattle (Michelizzi et al., 2010).

The Xilin buffalo is mainly produced in the plateau and mountainous areas of Xilin Longlin and Tianlin County of Guangxi. It is one of the local buffalo varieties in Guangxi. Due to the influence of natural ecological and environmental conditions and local socioeconomic activities, it is gradually formed after long -term natural selection and artificial selection. The Xilin buffalo is characterized by a large body size, gentle temperament, strong farming ability, good growth and development, efficient consumption of roughages, good mountain climbing, strong adaptability, and disease resistance (He et al., 2011). At the present, there is a single germplasm conservation farm in the main producing area which is primarily used for the production of hybrid females by crossing the local buffaloes with foreign excellent varieties (such as Murrah buffalo and Italian buffalo, mainly Murrah buffalo) for improvement of milk and meat production.

With the development of whole genome resequencing (WGS) technology, the reduction of sequencing cost, the genetic structure, evolutionary history, origin, and domestication of domestic animals such as pigs, cattle and sheep, etc. Have been widely and systematically studied become possible as an effective cost tool (Stothard et al., 2011).

Many WGS-based buffalo studies initially concentrated on the economically relevant characteristics of commercial breeds (Li et al., 2020). The genetic characteristics and selection pressure signals of Xilin buffalo have not been deeply studied by using WGS data earlier. The study on the genetic structure and population history of Xilin buffalo is helpful to analyze the genetic basis of adaptability and other traits and provides a theoretical basis for the improvement and conservation of Xilin buffalo varieties.

## 2 Materials and methods

### 2.1 Sample collection and sequencing

Blood and ear tissue samples were collected from the native home tract (Xilin County of Guangxi Province, China) of pure Xilin buffaloes (*n* = 25). The genomic DNA was extracted by the standard phenol-chloroform method (Green and Sambrook, 2012) and subjected to Illumina NovaSeq sequencing at Novogene Bioinformatics Institute, Beijing, China. By using pair-end sequencing technology an average insertion size of 500 bp was constructed for each sample and the average reading length was 150 bp. In addition, 46 published whole-genome sequences data of swamp buffalo including Guizhou white (*n* = 10), Binhu (*n* = 3), Fuzhong (*n* = 11), and Mediterranean (*n* = 22) were downloaded from NCBI(PRJNA547460) which fully described the characteristics of population structure, genetic diversity, single nucleotide polymorphisms (SNPs), and natural or artificial selection. The details of the five varieties are listed in Table 1.

**TABLE 1:** Sample information of 71 buffaloes from 5 buffalo breeds.

Varieties	Abbreviation	Sample size	Type
Xilin Buffalo	XL	25	Swamp
Guizhou White Buffalo	GZB	10	Swamp
Binhu buffalo	BH	3	Swamp
Fuzhong Buffalo	FZ	11	Swamp
Mediterranean Buffalo	MD	22	River

### 2.2 Construction of buffalo pseudo chromosome

In the present study, the published buffalo data were obtained from the reference genome assembly of buffalo (GCF_000471725.1) from NCBI. However, due to the complexity of the data, it is only assembled to the scaffold level. If it is directly used for comparison and subsequent analysis, it will lead to a double increase in computing and storage resources. Therefore, this study used the method of artificial connection of pseudo chromosomes. The reference genome is connected to 24 + X + unplaced chromosomes which can reflect the authenticity of chromosomes to the greatest extent (Amaral et al., 2008).

### 2.3 Genome wide alignment and variation detection

The sequenced reads after quality control were compared to the constructed buffalo pseudo chromosome by BWA-MEM (Li and Durbin, 2009), and repeated reads introduced by PCR were removed by Picard. The genome-wide high-quality genetic variation was detected by GATK (version 3.6-0-g89b7209) (Nekrutenko and Taylor, 2012) where the filtering conditions of SNP were as follows: (1) QD (Quality by Depth) < 2; (2) variants with FS (Phred-scaled *p*-value using Fisher’s exact test to detect strand bias) > 60 were filtered; MQRankSum (Z-score From Wilcoxon rank sum test of Alt vs. Ref read mapping qualities) < 12.5; (4) ReadPosRankSum (evaluate the reliability of variation by the position of variation in read) < −8; (5) MQ (RMS Mapping Quality) < 40.0; (6) Mean sequencing depth > 3x or < 1/3x (7) SOR (StrandOddsRatio) > 3.0; (8) maximum missing rate < .1; (9) SNP is strictly limited to double alleles. The Annovar software was used to annotate the variant information.

### 2.4 Analysis of buffalo population structure

First, VCF files of SNPs of 71 buffalo were converted into corresponding Plink files (bed. bim. fam by using vcftools) and PLINK (version 1.9) (Purcell et al., 2007) software were used to filter out the linkage disequilibrium sites with R2 greater than .2. The parameter is set as: -- indep pairwise 50 50.2. The filtered data were used to construct NJ tree, PCA, ADMIXTURE and other population structure-related analyses. In order to clarify, the phylogenetic relationship of 71 Buffalo, adjacency tree (NJ phylogenetic tree) (Yu et al., 2021) is constructed in this study. The genetic distance matrix is calculated by using the parameter “-- distance matrix” of PLINK 1.9 and then the matrix is transformed into. meg format, which will get imported .meg format into the MEGA6.0 software. Build the NJ phylogenetic tree and set the bootstrap value to 1000. Finally, using online iTOL (https://itol.embl.de/), the tool displays the obtained phylogenetic tree and beautifies it. The software package EIGENSOFT V5.0 and SmartPCA (Patterson et al., 2013) were used for PCA analysis of filtered buffalo autosomal SNP data sets. The significance of each eigenvector is calculated by the Tracy-Widom test. Admixture v. 1.3.0 (Alexander et al., 2009) was used to analyse the ancestral components of 71 buffalo autosomal SNP data sets. This study simulates that from k = 2 to k = 5. The bootstrap value of each k value was set to 20 and the optimal value was finally obtained according to the Cross-Validation (CV) value.

### 2.5 Genetic diversity, linkage disequilibrium and ROH detection

We used VCFtools to estimate the nucleotide diversity of each breed in window sizes of 50 kb with 50 kb increments. The Linkage disequilibrium (LD) decay with the physical distance between SNPs was calculated and visualized by using PopLDdecay software with default parameters (Rahimmadar et al., 2021). The run of homozygosity (ROH) was identified using the--homozyg option implemented in PLINK which slides a window of 50 SNPs (-homozyg-window-snp 50) across the genome estimating homozygosity (Makanjuola et al., 2021). The following settings were performed for ROH identification: (1) required minimum density (−homozyg-density 50); (2) number of heterozygotes allowed in a window (−homozyg-window-het 3); (3) the number of missing calls allowed in window (−homozyg-window-missing 5). The number and length of ROH for each breed were estimated and length of ROH was divided into three categories: .5–1 Mb, 1–2 Mb, 2–4 Mb. (Forutan et al., 2018). *F*_ROH_ is calculated by calculating the ratio of the total length of ROH fragments in the genome to the total length (*L*_ROH_) of the genome (*L*_auto_). The formula is as follows: *F*_ROH_ = ∑ *L*_ROH_/*L*_auto_

### 2.6 Selective scanning recognition

We adopted the following strategies for genome scanning of Xilin buffalo. First, we utilized nucleotide diversity (θπ) (Hudson, 1992) and the composite likelihood ratio test (CLR) (Nielsen et al., 2005) to detect the selection characteristics of Xilin buffalo. By using VCFtools, the nucleotide diversity was estimated using a sliding window of 50 kb and a step size of 20 kb. We used SweepFinder to calculate the CLR test of the sites in the non-overlapping 50 kb window in order to calculate the empirical *p*-value of π and CLR window and take the overlapping part of the first 1% window of each method as the candidate mark for selection.

Second, we performed comparisons between Xilin buffalo and Mediterranean buffalo using fixation index (*F*_*ST*_) (Hudson, 1992) and cross-group extended haplotype homozygosity (XP-EHH) (Sabeti et al., 2007). FST analysis was calculated in 50 kb windows with a 20 kb step using VCFtools (Danecek et al., 2011). XP-EHH statistics based on the extended haplotype was calculated for each population pair using selscan v1.1 (Szpiech and Hernandez, 2014). For XP-EHH selective scanning, our test statistic is the average normalized XP-EHH score of each 50 kb region. An XP-EHH score is directional: a positive score suggests that selection is likely to have happened in Xilin buffalo, whereas a negative score suggests the same about reference population. Significant genomic regions were identified by *p*-value < .01. Genomic regions identified by at least two methods were considered to be candidate regions of positive selection.

To better understand, the gene function and signaling pathways of the identified candidate genes, KOBAS 3.0 was used for GO and KEGG pathway enrichment analysis (Shen et al., 2019). Only when the corrected *p*-value < .05, were the GO and KEGG pathways considered significantly enriched.

## 3 Results

### 3.1 Identification of single nucleotide polymorphisms

In this study, individual genomes of 25 Xilin buffaloes were generated to ∼ 12.1 × coverage each and were jointly genotyped with publicly available genomes of three buffalo populations from different regions of China and Mediterranean Buffalo (Italy), and the average mapping rate was 99.37% (Supplementary Table S1). In total, ∼ 5.0 billion reads of sequences were generated. Using BWA-MEM, reads were aligned to the buffalo reference genome sequence (GCA_000471725.1) with an average of 10.6 × coverage. We annotated 28,347,965 biallelic SNPs found in 71 buffaloes. genomic annotation for showing the location of those SNPs that most of the SNPs existed in the intron region (65.532%) or intergenic region (19.514%). The exon contains merely 2.15% of the total SNPs with 529,920 synonymous SNPs (Supplementary Table S3).

### 3.2 Population genetic structure and genetic relationship

**FIGURE 1:**
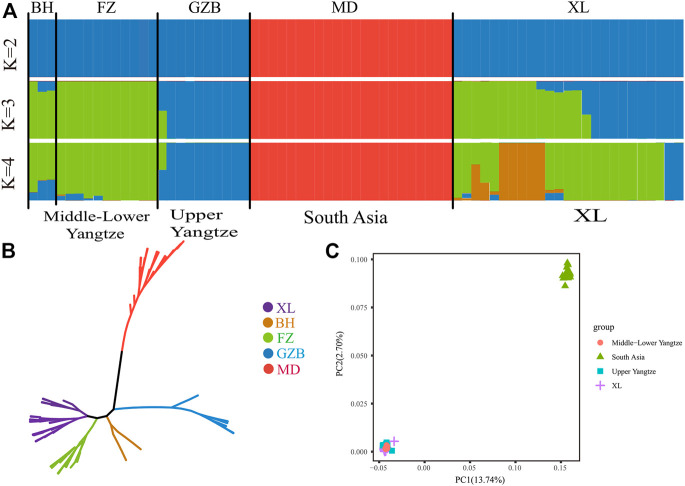
Population structure and relationships of Xinlin Buffalo. **(A)** Model-based clustering of buffalo using the ADMIXTURE program with K = 2 to 4(X). **(B)** Neighbour-joining tree of buffaloes constructed using whole-genome autosomal SNP data. **(C)** Principal component analysis (PCA) showing PC1 against PC2. The X axis represents PC1, and the Y axis represents PC2.

At present, there are ∼202 million buffaloes in the world, mainly distributed in Asia (196 million, accounting for 97.0%), Africa (3.4 million) and South America (∼2 million). (Zhang et al., 2007). According to the previous research of Sun et al. (2020), Asian buffaloes are divided into five regions according to their geographical distribution: the upper reaches of the Yangtze River, the middle and lower reaches of the Yangtze River, Southwest China, Southeast Asia and South Asia, and added Italy (Mediterranean buffalo). NJ phylogenetic tree was constructed from the whole genome data of 71 buffalo. As shown in (Figure 1A), the different colors represent buffaloes in different regions. These 71 buffalo are mainly divided into two branches: swamp and river buffalo. As for swamp buffalo is concerned, the buffalo in the same geographical area gather together. Some individuals are in the middle of the two types of buffalo in the phylogenetic tree which represents the hybrid individuals produced by the hybridization of the two types of buffalo. Principal component analysis (PCA) was used to further explore the genetic relationship between different buffalo populations. The results of PCA show that PC1(13.74%) and PC2(2.70%) distinguish riverine from swamp buffalo and the results of PC3(2.48%) show that Xilin buffalo is more similar to Middle lower Yangtze buffalo which is consistent with the literature (Figures 1B, C).

The whole genome data of 71 buffalo were analyzed by ADMIXTURE in order to perform ancestral component analysis (Figure 1A). When there is k = 2 it indicates the buffaloes of riverine and swamp origin. When there is k = 3, it shows that swamp buffaloes can be divided into two groups: Middle-Lower Yangtze buffalo (green) and Upper Yangtze buffalo (blue). When k = 4, it represented that the Xilin buffalo is classified as Yangtze River buffalo.

### 3.3 Genomic variation pattern

**FIGURE 2:**
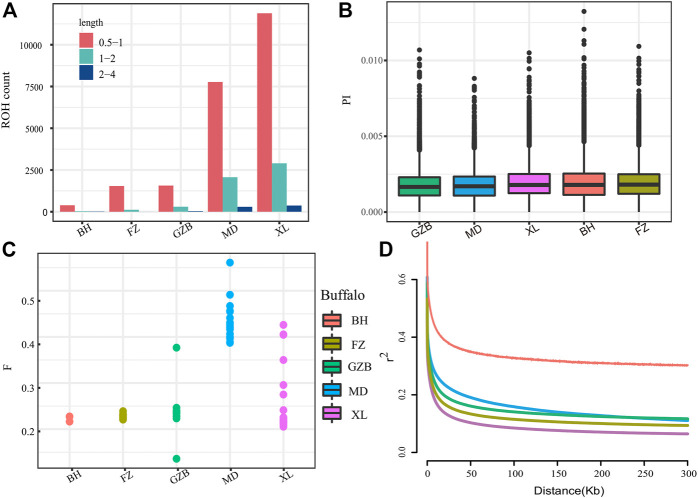
Summary statistics for genomic variation: **(A)** The distribution of the total number of ROH across chromosomes. **(B)**Genome-wide distribution of nucleotide diversity of each breed in 50 kb windows with 20 kb steps. **(C)** Inbreeding coefficient from each breed. **(D)** Genome-wide average LD decay estimated from each breed. The X axis is the physical distance (kb), and the Y axis is the LD coefficient (r^2^).

The runs of homozygosity (ROH) are a continuous homozygous region in the DNA sequence of diploid organisms. We used ROH to evaluate the homozygosity of each individual. To evaluate the ROH patterns of Xilin buffalo and other buffalo breeds, we divided the length of ROH into three categories: .5–1 Mb, 1–2 Mb, and 2–4 Mb. A long ROH is the result of blood mating, while a shorter ROH reflects the influence of distant ancestors. The identified ROH length was mostly between .5 and 1 Mb (ROH diagram) (Figure 2A). The π map showed that the nucleotide diversity of the Xilin buffalo was the highest, followed by that of the Binhu buffalo, Fuzhong buffalo, Mediterranean buffalo, and Guizhou white buffalo (Figure 2B). The inbreeding degree of the inbreeding population is usually measured by the average inbreeding coefficient of the population. The inbreeding coefficient refers to the degree of gene purification expressed as a percentage according to the number of generations of inbreeding. According to the results in the figure, the average locus of Mediterranean buffalo is the highest, indicating that the population was first and most stable through artificial breeding, and the inbreeding coefficient of other breeds is close (Figure 2C). The whole genome average linkage disequilibrium (LD) of the Xilin buffalo is the lowest, and the LD value of the Binhu buffalo is the highest. Due to the different genetic backgrounds of different populations with the same population type and species, the decay rate of LD is also very different. Domestication selection will reduce the genetic diversity of the population and strengthen the correlation (linkage degree) between loci. Therefore, in general, the higher the degree of domestication, the greater the selection intensity, and the slowest rate of LD attenuation (El et al., 2021) (Figure 2D).

### 3.4 Functional enrichment analysis of specific SNP in Xilin buffalo

**FIGURE 3:**
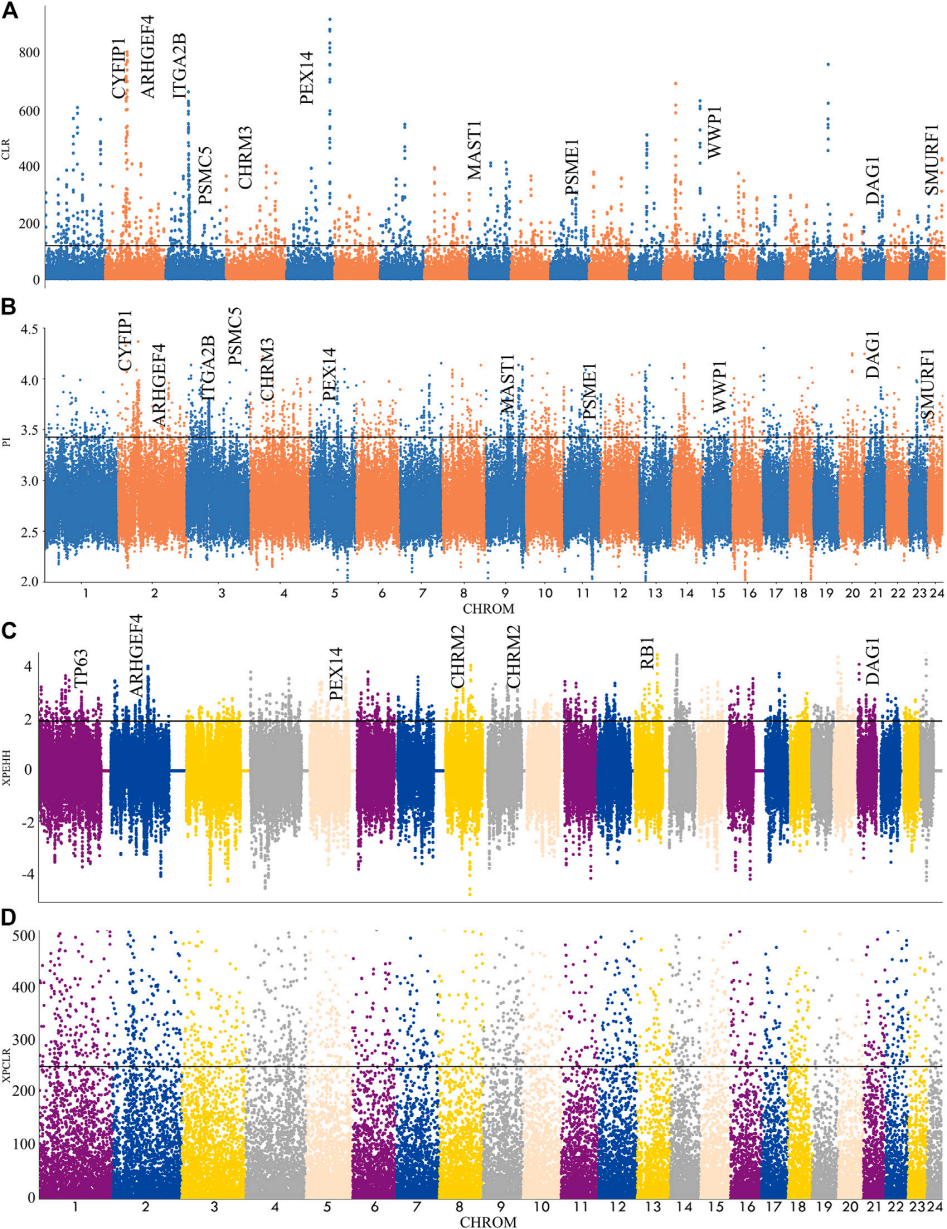
Genome-wide selection scan in Xilin buffalo using sliding window analysis (50 kb window size, 20 kb step size, 99th percentile cut-off). **(A)**. Selection signatures in Xilin buffalo for CLR (Mediterranean and Xilin). **(B)**. Selection signatures in Xilin buffalo for π-ratio (Mediterranean and Xilin). **(C)** Selection signatures in Xilin buffalo for XPEHH (Mediterranean and Xilin). **(D)** Selection signatures in Xilin buffalo for XPCLR (Mediterranean and Xilin). The thresholds (top 1%) of *F*_*ST*_, π-ratio, XPEHH and XPCLR are marked with a horizontal black line.

In this experiment, four methods (*F*_*ST*_, π ratio, XP-CLR, XP-EHH) were used to detect the selection signal of Xilin buffalo by comparing the Xilin buffalo population with the Mediterranean buffalo population (Figure 3). Among the four methods, if a gene was significantly detected by at least two methods (*p* < .005), the gene was regarded as a real candidate gene.

A total of 113 genes were screened and many KEGG pathways and Gene Ontology (GO) related to nerves and exercise endurance were significantly enriched (corrected *p*-value<.05). The KEGG pathway is significantly related to the nervous system with the glutamatergic synapse. The GO enrichment analysis detected many nerves and muscle-related GO entries, including ’Nervous system development, GO:007399′, ‘Neuronal projection, GO: 0043005’, ‘actin binding, GO:003779’, which reflect the nervous system and endurance played an extremely important role in the domestication and breeding of the Xilin buffalo.

### 3.5 Genome wide selective scanning test

**FIGURE 4:**
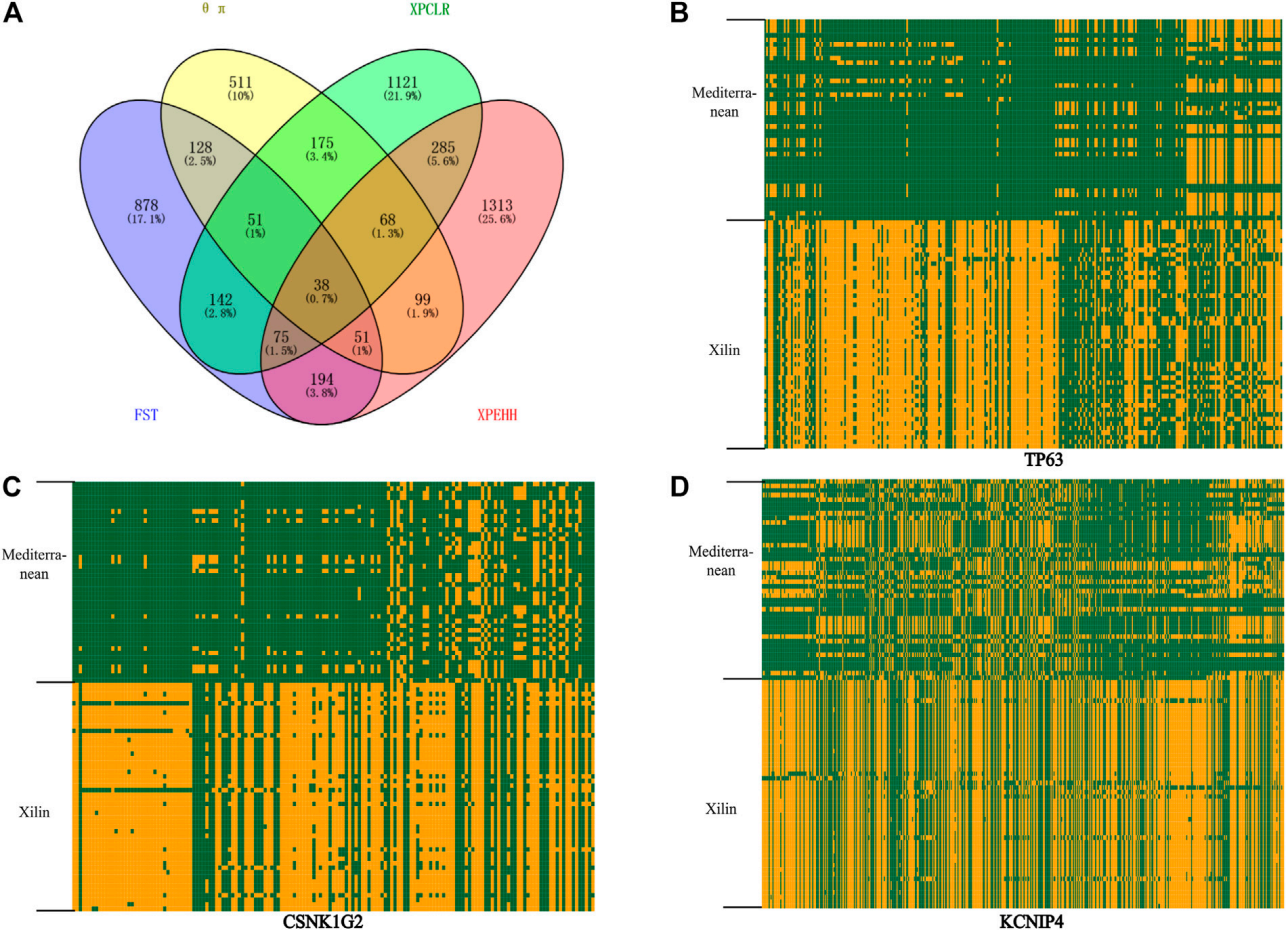
Analysis of the signatures of positive selection in the genome of Xilin Buffalo **(A)** Venn diagram showing the gene overlap among θπ, XPCLR, FST and XP-EHH. **(B)** Haplotype pattern heatmaps of the *TP63* gene region. **(C)** Haplotype pattern heatmaps of the *CSNK1G2* gene region. **(D)** Haplotype pattern heatmaps of the *KCNIP4* gene region. The major allele at each SNP position in Xilin buffalo is colored in yellow, the minor one in green.

Nucleotide diversity analysis (θ π) and complex likelihood ratio (CLR) were used to detect the selection-related genomic regions in the Xilin buffalo population. A total of 1121 (θ π) and 677 (CLR) (Figure 4A) genes were identified in Xilin buffalo with 357 overlapped. One of the most significant pathways (*p*-value < .05) was the Regulation of actin cytoskeleton which contained five genes (*CYFIP1, ITGA2B, ARHGEF4, CHRM3, CHRM2*) related to beef tenderness, feed efficiency and compensatory gain (Xia et al., 2021). Based on the gene ontology analysis of Xilin Buffalo, it is found that Xilin Buffalo has increased the GO category, including ‘microtubule anchoring’ (*MAST1, DAG1, PEX14*), ‘Proteasome mediated ubiquitin-dependent protein catabolism’ (*SMURF1, WWP1, PSME2, PSME1, DCAF11, HERC2, PSMC5*).

*F*_*ST*_ and XP-EHH tests were used to detect the positive selection characteristics of Xilin and riverine (Mediterranean) buffaloes Through the analysis, 1557 and 2123 hypothetical favorable positive selection genes were obtained from *F*_*ST*_ and XP-EHH methods, respectively, and 358 genes were obtained from both methods.

38 overlapping genes were detected in the above four selection methods which indicates that these genes have strong selection ability in the Xilin buffalo (Figure 4A). It is worth noting that (*CSNK1G2, TP63*), *CSNK1G2* is related to spermatogenesis, *MFG-E8* is a sign of high milk production in dairy animals, *TP63* participates in breast secretion by activating *MFG-E8*, and *RB1* is related to the formation of bovine intramuscular fat (marbling) (Lim et al., 2013).

## 4 Discussion

Understandings the characteristics of population structure and genetic diversity is very important for genetic evaluation, environmental adaptation, utilization, and protection of genetic resources of cattle breeds. In the present study, the whole genome sequences of 25 Xilin buffaloes were analysed. According to the geographical distribution, the buffalo are divided into six geographical regions: Upper Yangtze, middle lower Yangtze, Southwest China, Southeast Asia, South Asia, and Italy. Through ADMIXTURE analysis, we proved that the Xilin buffalo belongs to the Yangtze River.

The nucleotide diversity level of Xilin buffalo was slightly higher than the other breeds (average θπ = .0017). The relatively high genomic diversity of Xilin buffalo might be related to its weak and short selection history. The Xilin buffalo showed a similar structural heritability to Fuzhong Buffalo which is related to its similar geographical location and genetic background. In addition, the LD attenuation pattern of each variety was basically consistent with the results of nucleotide diversity. The ROH distribution pattern of Xilin buffalo was analyzed by comparing it with other cattle breeds. The ROH is common in bovine autosomes but the observed varietal differences in ROH length and burden patterns indicate differences in varietal origin and recent management. Compared with the cattle breeds analyzed in this study, Xilin buffalo showed more short/medium ROH (.5–2 Mb) and the average number of ROH was the highest.

By comparing with Mediterranean buffalo, we found that Xilin buffalo has unique signaling pathways in the nervous system, reproductive system, and lactation. In this study, the KEGG pathway and GO related to the nervous system were significantly enriched and the most significantly enriched pathway was the Glutamatergic synapse. In addition, GO analysis is also significantly enriched by many GO items related to the development of the nervous system such as neurons, dendritic spines, and synapses, and positive regulation of dendrite morphogenesis. Previous studies have proved that dendritic spines and their structural and functional plasticity are the cellular basis of learning and memory (Kasai et al., 2003). Therefore, it is speculated that these neural-related KEGG pathways and GO entries also play an extremely important role in the domestication of swamp buffalo. It has been reported that the glutamatergic synaptic pathway is related to the adaptability of mice to stress and fear behavior (Kamprath et al., 2010). It contains three genes *GRM5*, *GRIK2,* and *GRIA4,* and *GluR6* is encoded by *GRIK2* which is highly expressed in the brain and is associated with autosomal recessive intellectual disability (Motazacker et al., 2007). The *GRIK2* knockout mice showed decreased fear and memory, anxiety, and despair (Shaltiel et al., 2008). In rabbits, *GRIK2* was identified as a candidate domestication gene (Carneiro et al., 2014). The *GRIK2* is highly expressed in the brain tissues of buffalo, goats, sheep, and cattle. Studies have reported that *GRM5* is related to social interaction and sports behavior (Xu et al., 2021). The swamp buffalo has a gentle temperament and is mainly used for servitude. It can be easily trained for rice farming, cart pulling, and other labor (Chantalakhana and Bunyavejchewin, 1994). These traits indicate that the identified pathways and candidate genes related to the nervous system were strongly artificially selected during the domestication of swamp buffalo.

Reproductive performance is an important index to measure the economic benefit of a variety. The Xilin Buffalo has good reproductive performance and its oestrus cycle is between 20 and 25 days, with an average of 21.04 days. We found that both *CSNK1G2* and *KCNIP4* genes showed universal strong positive selection signals in Xilin buffalo/Mediterranean buffalo and both were related to reproduction. The *CSNK1G2* gene is related to sperm surface modification, sperm maturation, and sperm-egg communication of bull sperm (Byrne et al., 2012). The *CSNK1G2* gene is associated to the ability of frozen-thawed sperm to respond appropriately to stress (Pini et al., 2018). It has also been observed that *CSNK1G2* knockout mice show premature aging of the testes (Li et al., 2020) whereas the *KCNIP4* gene is closely related to chicken reproductive traits (Fan et al., 2017).

The Xilin buffalo was mainly used for both meat and milk. After crossbreeding, the introduced milk variety Mora buffalo over the years, the average lactation yield of the three generations has increased significantly (2389 ± 700.2 kg) and now its value is progressing because of better milk and meat production. In this study, we also found a selection signal related to lactation (*TP63*) and *MFG-E8* as a marker of high milk production in dairy animals. The *TP63* participates in breast secretion by activating *MFG-E8*. Previous studies have confirmed that *TP63* plays a role in regulating the growth and differentiation of mammary epithelial cells (Verma et al., 2021). By considering the influence of natural ecological environmental conditions and local social and economic activities, these genes may play an important role in the reproduction and lactation performance of the Xilin buffalo after long-term natural and artificial selection.

## 5 Conclusion

Utilizing WGS data, the present study described the Xilin buffalo’s whole genome level. The direction for the genetic assessment and coherent breeding plan of the Xilin buffalo was identified by examining the characteristics of population structure and genomic diversity. In addition, we also identified a series of candidate genes involved in milk production, neural control, and fertility. Moreover, the results of this study enable breeders to better understand the genomic characteristics of Xilin buffalo for artificial selection or adaptation to the local environment.

## Data Availability

The datasets presented in this study can be found in online repositories. The names of the repository/repositories and accession number(s) can be found below: https://www.ncbi.nlm.nih.gov/, PRJNA573503.

## References

[B1] Alexander D. H. Novembre J. Lange K. (2009). Fast model-based estimation of ancestry in unrelated individuals. Genome Res. 19 (9), 1655–1664. 10.1101/gr.094052.109 19648217 PMC2752134

[B2] Amaral M. E. J. Grant J. R. Riggs P. K. Stafuzza N. B. Womack J. E. Goldammer T. (2008). A first generation whole genome RH map of the river buffalo with comparison to domestic cattle. Bmc Genomics 9, 631. 10.1186/1471-2164-9-631 19108729 PMC2625372

[B3] Byrne K. Leahy T. McCulloch R. Colgrave M. L. Holland M. K. (2012). Comprehensive mapping of the bull sperm surface proteome. Proteomics 12 (23-24), 3559–3579. 10.1002/pmic.201200133 23081703

[B4] Carneiro M. Rubin C. J. Di Palma F. Albert F. W. Alfoldi J. Martinez B. A. (2014). Rabbit genome analysis reveals a polygenic basis for phenotypic change during domestication. Science 345 (6200), 1074–1079. 10.1126/science.1253714 25170157 PMC5421586

[B5] Chantalakhana C. Bunyavejchewin P. (1994). Buffaloes and draught power. Outlook Agric. 23 (2), 91–95. 10.1177/003072709402300204

[B6] Danecek P. Auton A. Abecasis G. Albers C. A. Banks E. DePristo M. A. (2011). The variant call format and VCFtools. Bioinformatics 27 (15), 2156–2158. 10.1093/bioinformatics/btr330 21653522 PMC3137218

[B7] Di Stasio L. Brugiapaglia A. (2021). Current knowledge on River buffalo meat: A critical analysis. Anim. (Basel) 11 (7), 2111. 10.3390/ani11072111 PMC8300413 34359238

[B8] El H. A. Rocha D. Venot E. Blanquet V. Philippe R. (2021). Long-range linkage disequilibrium in French beef cattle breeds. Genet. Sel. Evol. 53 (1), 63. 10.1186/s12711-021-00657-8 34301193 PMC8306006

[B9] Fan Q. C. Wu P. F. Dai G. J. Zhang G. X. Wang J. Y. Xue Q. (2017). Identification of 19 loci for reproductive traits in a local Chinese chicken by genome-wide study. Genet. Mol. Res. Gmr 16 (1). 10.4238/gmr16019431 28340264

[B10] Fischer H. Ulbrich F. (1967). Chromosomes of the Murrah buffalo and its crossbreds with the asiatic swamp buffalo (Bubalus bubalis). J. Animal Breed. Genet. 84 (1-4), 110–114. 10.1111/j.1439-0388.1967.tb01102.x

[B11] Forutan M. Ansari M. S. Baes C. Melzer N. Schenkel F. S. Sargolzaei M. (2018). Inbreeding and runs of homozygosity before and after genomic selection in North American Holstein cattle. BMC Genomics 19 (1), 98. 10.1186/s12864-018-4453-z 29374456 PMC5787230

[B12] Green M. R. Sambrook J. (2012). “Molecular cloning: A laboratory manual,” in Three-volume set. Fourth Edition (United States: Cold Spring Harbor Laboratory Pr).

[B13] He L. Su J. Zhang L. Deng S. He Y. Huang X. (2011). Survey on national local breeds - Fuzhong and Xilin buffalo. Guangxi Animal Husb. Veterinary Med. 27 (6), 3. (In Chinese).

[B14] Hudson R. (1992). Estimation of levels of gene flow from DNA sequence data. Genetics 132, 583. 10.1093/genetics/132.2.583 1427045 PMC1205159

[B15] Kamprath K. Plendl W. Marsicano G. Deussing J. M. Wurst W. Lutz B. (2010). Endocannabinoids mediate acute fear adaptation via glutamatergic neurons independently of corticotropin-releasing hormone signaling. Genes Brain & Behav. 8 (2), 203–211. 10.1111/j.1601-183X.2008.00463.x 19077175

[B16] Kasai H. Matsuzaki M. Noguchi J. Yasumatsu N. Nakahara H. (2003). Structure–stability–function relationships of dendritic spines. Trends Neurosci. 26 (7), 360–368. 10.1016/S0166-2236(03)00162-0 12850432

[B17] Li D. Ai Y. Guo J. Dong B. Wang X. (2020). Casein kinase 1G2 suppresses necroptosis-promoted testis aging by inhibiting receptor-interacting kinase 3. eLife Sci. 9, e61564. 10.7554/eLife.61564 PMC7673785 33206046

[B18] Li H. Durbin R. (2009). Fast and accurate short read alignment with Burrows–Wheeler transform. Bioinformatics 25, 1754. 10.1093/bioinformatics/btp324 19451168 PMC2705234

[B19] Li X. Yang J. Shen M. Xie X. L. Liu G. J. Xu Y. X. (2020). Whole-genome resequencing of wild and domestic sheep identifies genes associated with morphological and agronomic traits. Nat. Commun. 11 (1), 2815. 10.1038/s41467-020-16485-1 32499537 PMC7272655

[B20] Lim D. Lee S. H. Kim N. K. Cho Y. M. Kim H. Seong H. H. (2013). Gene Co-expression analysis to characterize genes related to marbling trait in hanwoo (Korean) cattle. Asian-australasian J. Animal Sci. 26 (1), 19–29. 10.5713/ajas.2012.12375 PMC4093059 25049701

[B21] Liu J. J. Liang A. X. Campanile G. Plastow G. Zhang C. Wang Z. (2018). Genome-wide association studies to identify quantitative trait loci affecting milk production traits in water buffalo. J. Dairy Sci. 101 (1), 433–444. 10.3168/jds.2017-13246 29128211

[B22] Makanjuola B. O. Maltecca C. Miglior F. Marras G. Abdalla E. A. Schenkel F. S. (2021). Identification of unique ROH regions with unfavorable effects on production and fertility traits in Canadian Holsteins. Genet. Sel. Evol. 53 (1), 68. 10.1186/s12711-021-00660-z 34461820 PMC8406729

[B23] Michelizzi V. N. Dodson M. V. Pan Z. Amaral M. E. J. Michal J. J. Mclean D. J. (2010). Water buffalo genome science comes of age. Int. J. Biol. Sci. 6 (4), 333–349. 10.7150/ijbs.6.333 20582226 PMC2892297

[B24] Motazacker M. M. Rost B. R. Hucho T. Garshasbi M. Kahrizi K. Ullmann R. (2007). A defect in the ionotropic glutamate receptor 6 gene (GRIK2) is associated with autosomal recessive mental retardation. Am. J. Hum. Genet. 81 (4), 792–798. 10.1086/521275 17847003 PMC2227928

[B25] Nekrutenko A. Taylor J. (2012). Next-generation sequencing data interpretation: Enhancing reproducibility and accessibility. Nat. Rev. Genet. 13 (9), 667–672. 10.1038/nrg3305 22898652

[B26] Nielsen R. Williamson S. Kim Y. Hubisz M. J. Clark A. G. Bustamante C. (2005). Genomic scans for selective sweeps using SNP data. Genome Res. 15 (11), 1566–1575. 10.1101/gr.4252305 16251466 PMC1310644

[B27] Patterson N. Price A. L. Reich D. (2013). Population structure and eigenanalysis. Plos Genet. 2 (12), e190. 10.1371/journal.pgen.0020190 PMC1713260 17194218

[B28] Pini T. Rickard J. P. Leahy T. Crossett B. Graaf S. de Graaf S. P. (2018). Cryopreservation and egg yolk medium alter the proteome of ram spermatozoa. J. Proteomics 181, 73–82. 10.1016/j.jprot.2018.04.001 29627624

[B29] Purcell S. Neale B. Todd-Brown K. Thomas L. Ferreira M. Bender D. (2007). Plink: A tool set for whole-genome association and population-based linkage analyses. Am. J. Hum. Genet. 81 (3), 559–575. 10.1086/519795 17701901 PMC1950838

[B30] Rahimmadar S. Ghaffari M. Mokhber M. Williams J. L. (2021). Linkage disequilibrium and effective population size of buffalo populations of Iran, Turkey, Pakistan, and Egypt using a medium density SNP array. Front. Genet. 12, 12608186. 10.3389/fgene.2021.608186 PMC8689148 34950186

[B31] Sabeti P. C. Varilly P. Fry B. Lohmueller J. Hostetter E. Cotsapas C. (2007). Genome-wide detection and characterization of positive selection in human populations. Nature 449, 449913–449918. 10.1038/nature06250 PMC2687721 17943131

[B32] Shaltiel G. Maeng S. Malkesman O. Pearson B. Schloesser R. J. Tragon T. (2008). Evidence for the involvement of the kainate receptor subunit GluR6 (GRIK2) in mediating behavioral displays related to behavioral symptoms of mania. Mol. Psychiatry 13 (9), 858–872. 10.1038/mp.2008.20 18332879 PMC2804880

[B33] Shen S. Kong J. Qiu Y. Yang X. Wang W. Yan L. (2019). Identification of core genes and outcomes in hepatocellular carcinoma by bioinformatics analysis. J. Cell Biochem. 120 (6), 10069–10081. 10.1002/jcb.28290 30525236

[B34] Stothard P. Choi J. W. Basu U. Sumner-Thomson J. M. Meng Y. Liao X. (2011). Whole genome resequencing of black Angus and Holstein cattle for SNP and CNV discovery. BMC Genomics 12, 559. 10.1186/1471-2164-12-559 22085807 PMC3229636

[B35] Sun T. Shen J. Achilli A. Chen N. Chen Q. Dang R. (2020). Genomic analyses reveal distinct genetic architectures and selective pressures in buffaloes. Gigascience 9 (2), giz166. 10.1093/gigascience/giz166 32083286 PMC7033652

[B36] Szpiech Z. A. Hernandez R. D. (2014). selscan: an efficient multithreaded program to perform EHH-based scans for positive selection. Mol. Biol. Evol. 31 (10), 2824–2827. 10.1093/molbev/msu211 25015648 PMC4166924

[B37] Verma A. K. Ali S. A. Singh P. Kumar S. Mohanty A. K. (2021). Transcriptional repression of MFG-E8 causes disturbance in the homeostasis of cell cycle through DOCK/ZP4/STAT signaling in buffalo mammary epithelial cells. Front. Cell Dev. Biol. 9, 568660. 10.3389/fcell.2021.568660 33869165 PMC8047144

[B38] Xia X. Zhang S. Zhang H. Zhang Z. Chen N. Li Z. (2021). Assessing genomic diversity and signatures of selection in Jiaxian Red cattle using whole-genome sequencing data. BMC Genomics 22 (1), 43. 10.1186/s12864-020-07340-0 33421990 PMC7796570

[B39] Xu J. Marshall J. J. Kraniotis S. Nomura T. Zhu Y. Contractor A. (2021). Genetic disruption of Grm5 causes complex alterations in motor activity, anxiety and social behaviors. Behav. Brain Res. 411, 411113378. 10.1016/j.bbr.2021.113378 PMC8238894 34029630

[B40] Yu Z. Zhang W. Gu C. Chen J. Zhao M. Fu L. (2021). Genomic analysis of Ranavirus and exploring alternative genes for phylogenetics. Transbound. Emerg. Dis. 68 (4), 2161–2170. 10.1111/tbed.13864 33006817

[B41] Zhang Y. Sun D. Yu Y. (2007). Genetic diversity and differentiation of Chinese domestic buffalo based on 30 microsatellite markers. Anim. Genet. 38, 569. 10.1111/j.1365-2052.2007.01648.x 17980000

